# Persistent Methicillin-Resistant Staphylococcus aureus (MRSA) Bacteremia Due to Pelvic Pyomyositis and Abscesses With Extension Through the Sciatic Notch: A Case Report

**DOI:** 10.7759/cureus.117021

**Published:** 2026-09-27

**Authors:** Nastaran Biriji, Dylan Mooney, Andrew Hamilton

**Affiliations:** 1 Family Medicine, Henry Ford Genesys Hospital, Henry Ford Health, Grand Blanc, USA

**Keywords:** deep pelvic pyomyositis, gluteal abscess, intramuscular abscess, mri, mrsa, obturator internus, pelvic abscess, persistent bacteremia, sciatic nerve, sciatic notch

## Abstract

Pelvic pyomyositis can present a diagnostic challenge because of its anatomic location and nonspecific clinical manifestations. We report a case of a 39-year-old man with poorly controlled diabetes mellitus with neuropathy and bilateral foot ulcers who presented with 10 days of persistent methicillin-resistant *Staphylococcus aureus* (MRSA) bacteremia and progressive left hip pain. Transthoracic and transesophageal echocardiography and computed tomography (CT) of the abdomen and pelvis did not identify a definitive source of the persistent bacteremia. Contrast-enhanced magnetic resonance imaging (MRI) of the pelvis subsequently demonstrated multifocal intramuscular abscesses involving the obturator internus, gluteus minimus, and gluteus medius muscles, with extension through the greater sciatic notch and along the sciatic nerve. MRI established the diagnosis of multifocal deep pelvic pyomyositis. Surgical and percutaneous drainage were not considered feasible. The patient was treated with prolonged intravenous daptomycin, with subsequent clearance of bacteremia and clinical improvement. This case demonstrates an advanced stage of pelvic and sciatic infection involving the greater sciatic notch, highlighting the value of MRI in identifying and delineating a musculoskeletal source of persistent MRSA bacteremia when initial imaging was inconclusive.

## Introduction

Pyomyositis is a primary bacterial infection of skeletal muscle characterized by suppurative inflammation and abscess formation [1,2]. Although historically associated with tropical regions, its incidence has increased in temperate climates, particularly among patients with diabetes mellitus, immunosuppression, chronic skin infections, chronic kidney disease, and intravenous drug use [3-5]. A nationwide study of pyomyositis admissions in the United States from 2002 to 2014 demonstrated a more than three-fold increase in incidence, with significant associations with diabetes mellitus, HIV, and chronic kidney disease [4]. *Staphylococcus aureus*, including methicillin-resistant *S. aureus* (MRSA), remains the predominant causative organism [1,6].

Clinical manifestations vary with the location and stage of infection and commonly include fever, localized muscle pain, tenderness, and limitation of movement; however, deep muscle involvement may have few external findings and can delay diagnosis [2,3,7]. Differential diagnoses include septic arthritis, osteomyelitis, hematoma, muscle injury, and other infectious or inflammatory musculoskeletal conditions [3,7]. Diagnosis is based on clinical findings, microbiologic cultures, and imaging, with MRI being the preferred modality for identifying muscle inflammation, intramuscular abscesses, and involvement of adjacent structures [1,7]. Management consists of appropriate antimicrobial therapy and drainage of purulent collections when safely feasible [1,3,7]. Delayed or inadequately controlled infection may result in persistent bacteremia, sepsis, metastatic infection, osteomyelitis, or septic arthritis [3,7].

We present a 39-year-old man with poorly controlled type 2 diabetes mellitus complicated by neuropathy and chronic bilateral foot ulcers who developed persistent MRSA bacteremia and progressive left hip pain. MRI of the pelvis demonstrated deep pelvic pyomyositis with intramuscular abscesses involving the obturator internus, gluteus minimus, and gluteus medius muscles, with extension through the greater sciatic notch and inflammatory changes along the sciatic nerve. This case highlights the value of MRI in identifying an occult deep pelvic infectious focus in a patient with persistent MRSA bacteremia.

## Case presentation

A 39-year-old man with poorly controlled type 2 diabetes mellitus complicated by peripheral neuropathy and chronic diabetic foot ulcers, hypertension, hyperlipidemia, remote intravenous drug use, and housing insecurity presented with a 10-day history of fever, chills, dysuria, urinary frequency, and progressive left hip pain radiating down the posterior left leg. He also reported intermittent rectal pain and had been nonadherent to his medications because of loss of insurance coverage. On presentation, his temperature was 103.2°F, heart rate 119 beats/min, respiratory rate 30 breaths/min, and blood pressure 173/93 mmHg. He appeared ill and diaphoretic, with suprapubic and left lateral hip tenderness. Passive range of motion of the left hip was preserved without significant pain. Lower-extremity strength was preserved without focal motor deficits, with decreased distal sensation consistent with known diabetic peripheral neuropathy. Distal pulses were palpable bilaterally. Chronic ulcerations were present at the right transmetatarsal amputation stump and left first toe amputation site, without surrounding erythema, fluctuance, or purulent drainage. Laboratory evaluation demonstrated leukocytosis, with a white blood cell count of 21.3×10³/µL (Table 1). The comprehensive metabolic panel showed glucose 553 mg/dL, potassium 3.1 mmol/L, and total carbon dioxide 18 mmol/L (Table 2). Additional laboratory evaluation demonstrated a hemoglobin A1c of 11.7% and serum lactate of 2.3 mmol/L (Table 3). Urinalysis demonstrated pyuria and hematuria with negative urine ketones (Table 4).

**Table 1:** Complete blood count during the initial evaluation.

Laboratory parameters	Patient value	Reference range
White blood cell count	21.31×10³/µL	4.0-11.0×10³/µL
Red blood cell count	4.65×10⁶/µL	4.5-5.9×10⁶/µL
Hemoglobin	14.2 g/dL	13.5-17.5 g/dL
Hematocrit	40.1%	41-53%
Mean corpuscular volume	86.2 fL	80-100 fL
Mean corpuscular hemoglobin	30.5 pg	27-33 pg
Mean corpuscular hemoglobin concentration	35.4 g/dL	32-36 g/dL
Red cell distribution width	12.8%	11.5-14.5%
Platelet count	156×10³/µL	150-450×10³/µL

**Table 2:** Comprehensive metabolic panel findings during the initial evaluation.

Laboratory parameter	Patient value	Reference range
Sodium	135 mmol/L	135-145 mmol/L
Potassium	3.1 mmol/L	3.5-5.0 mmol/L
Chloride	106 mmol/L	98-107 mmol/L
Total carbon dioxide	18 mmol/L	22-29 mmol/L
Blood urea nitrogen	12 mg/dL	7-20 mg/dL
Creatinine	0.94 mg/dL	0.7-1.3 mg/dL
Glucose	553 mg/dL	70-99 mg/dL
Calcium	8.1 mg/dL	8.5-10.5 mg/dL
Total protein	6.2 g/dL	6.0-8.3 g/dL
Albumin	3.3 g/dL	3.5-5.0 g/dL
Alkaline phosphatase	138 U/L	44-147 U/L
Aspartate aminotransferase	26 U/L	10-40 U/L
Alanine aminotransferase	25 U/L	7-56 U/L
Total bilirubin	0.4 mg/dL	0.1-1.2 mg/dL

**Table 3:** Additional pertinent laboratory findings.

Laboratory parameter	Patient value	Reference range
Hemoglobin A1c	11.7%	4.0-5.6%
Serum lactate	2.3 mmol/L	0.5-2.2 mmol/L

**Table 4:** Urinalysis findings during the initial evaluation. HPF: high-power field

Urinalysis parameters	Patient result
Color	Yellow
Clarity	Cloudy
Specific gravity	1.033
pH	5.0
Protein	2+ (100 mg/dL)
Glucose	3+
Ketones	Negative
Blood	3+
Nitrite	Negative
Leukocyte esterase	Small
White blood cells	51-100/HPF
White blood cell clumps	Present
Red blood cells	11-25/HPF
Squamous epithelial cells	Few
Mucus threads	Few

Hyperglycemia was managed with basal and short-acting insulin, including correctional sliding-scale insulin, with no evidence of diabetic ketoacidosis or hyperosmolar hyperglycemic state. Blood cultures grew methicillin-resistant *Staphylococcus aureus* (MRSA), with positive cultures obtained on admission and again on hospital days four, seven, and 10. Antimicrobial susceptibility testing (AST) of the initial MRSA blood isolate demonstrated susceptibility to vancomycin (minimum inhibitory concentration {MIC} 1 µg/mL), clindamycin, erythromycin, tetracycline, and trimethoprim-sulfamethoxazole, with resistance to oxacillin, penicillin, cefazolin, cefuroxime, and amoxicillin-clavulanate (Table 5). Daptomycin susceptibility was not reported on the available AST panel. Urine culture also grew MRSA. Wound cultures from both feet grew MRSA, with *Escherichia coli* additionally isolated from the right foot ulcer. Podiatry debrided both chronic foot ulcerations and noted no clinical signs of acute infection requiring surgical intervention. Radiographs of both feet showed no findings suggestive of osteomyelitis. Left hip radiography on hospital day three showed no acute osseous abnormality. The patient was initially treated with intravenous vancomycin, with cefazolin added following the right foot wound culture results. Transthoracic and transesophageal echocardiography showed no evidence of infective endocarditis. Initial CT of the abdomen and pelvis on hospital day 2 demonstrated bladder and rectal wall thickening without a definitive deep musculoskeletal infectious focus. Because MRSA bacteremia persisted and left hip pain worsened, vancomycin was discontinued on hospital day eight, and intravenous daptomycin and cefazolin were continued per Infectious Disease team recommendations. MRI of the left hip on hospital day 10 demonstrated multiple intramuscular abscesses involving the obturator internus, gluteus minimus, and gluteus medius muscles, with extension through the greater sciatic notch and inflammatory changes along the sciatic nerve (Figure 1). No pelvic osteomyelitis or hip joint involvement was reported. MRI of the lumbar spine demonstrated degenerative disc disease without evidence of an infectious process. General surgery and interventional radiology determined that the pelvic collections were not safely amenable to operative or percutaneous drainage. Blood cultures became negative on hospital day 11 and remained negative thereafter. Cefazolin was discontinued on hospital day 14. Repeat CT of the abdomen and pelvis on hospital day 15 demonstrated an ill-defined, peripherally enhancing collection posterior to the left acetabulum with surrounding inflammatory changes (Figure 2). The patient subsequently demonstrated clinical improvement and was discharged on hospital day 18, with daily outpatient intravenous daptomycin infusions planned to complete approximately seven weeks of antimicrobial therapy under Infectious Disease team supervision. Figure 3 summarizes the chronology of the patient's diagnostic evaluation, persistent MRSA bacteremia, antimicrobial management, and subsequent clinical course.

**Figure 1:**
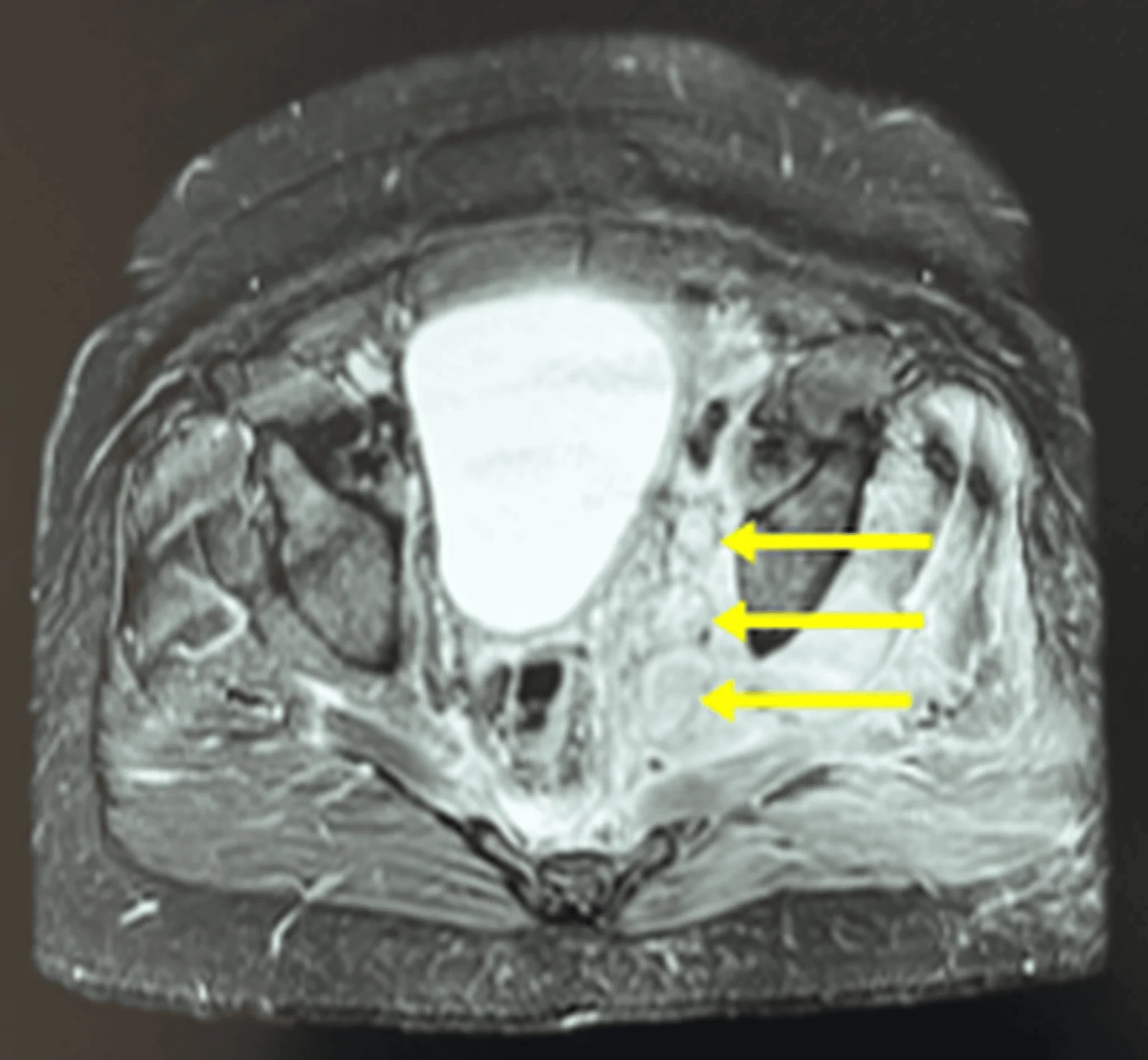
Axial contrast-enhanced MRI of the pelvis demonstrating multiple deep pelvic abscesses. Axial contrast-enhanced MRI demonstrates multiple rim-enhancing deep pelvic abscesses (yellow arrows) with surrounding soft-tissue inflammation and edema. The collections are located in close proximity to critical neurovascular structures, contributing to the difficulty of safe surgical or percutaneous drainage.

**Figure 2:**
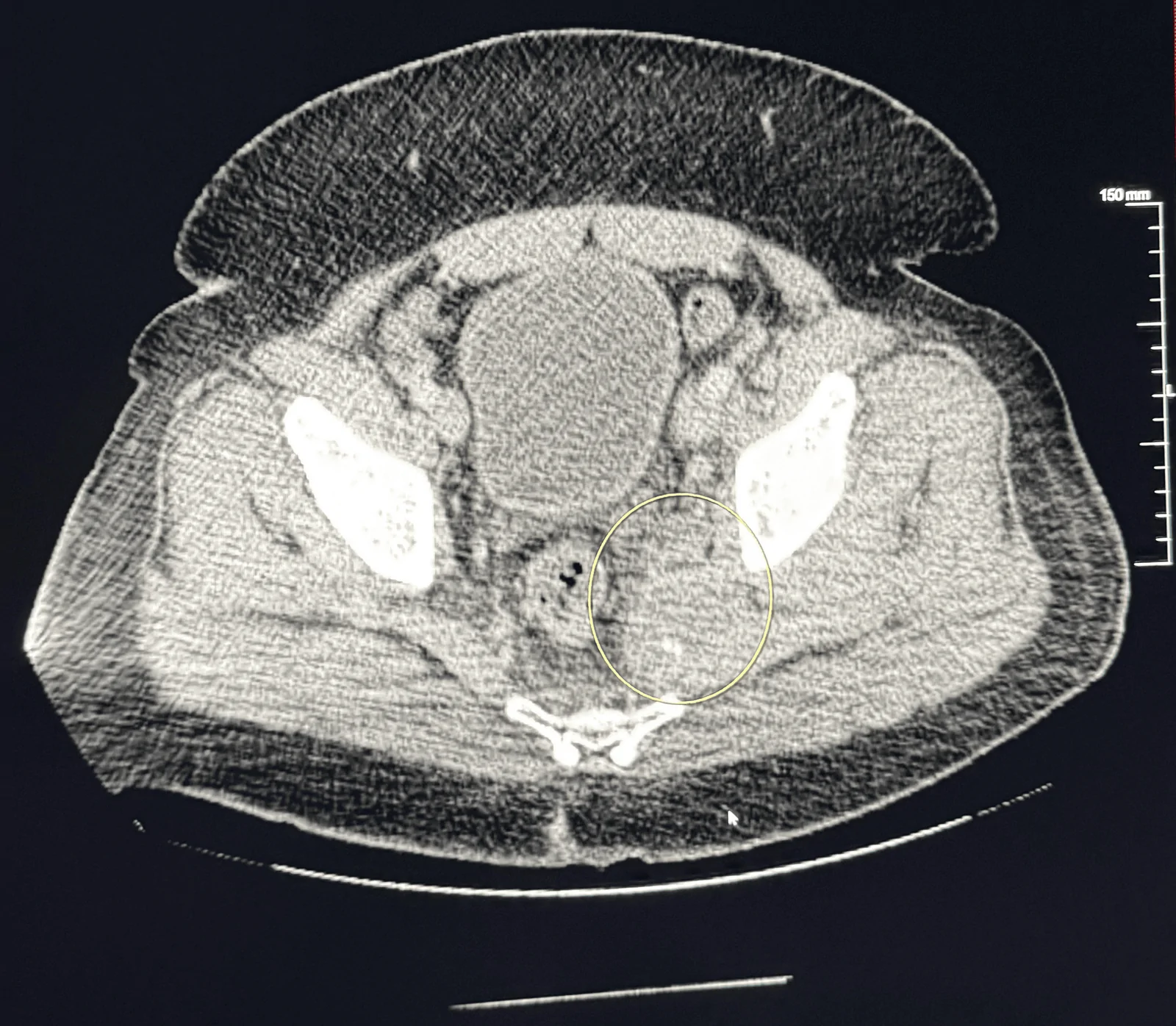
Contrast-enhanced CT demonstrating the deep pelvic infectious process. (A) Axial contrast-enhanced CT image demonstrating an ill-defined, peripherally enhancing collection adjacent to the posterior left acetabulum with surrounding inflammatory changes (circled).

**Figure 3:**
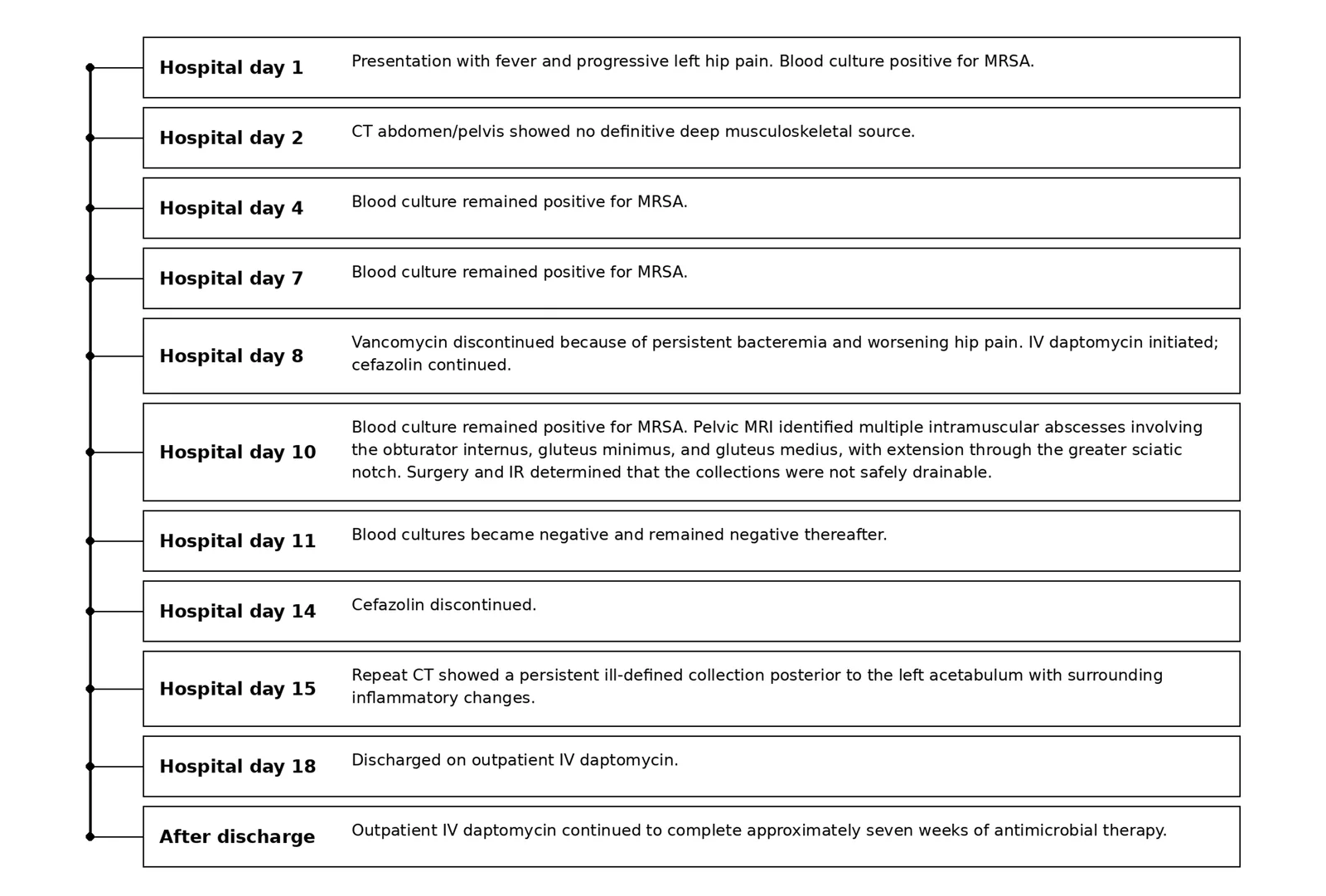
Timeline of clinical course, diagnostic evaluation, and management. Chronology of the patient's persistent MRSA bacteremia, diagnostic evaluation, identification of deep pelvic pyomyositis, antimicrobial management, clearance of blood cultures, and subsequent outpatient treatment. MRSA: methicillin-resistant *Staphylococcus aureus*; IR: interventional radiology

**Table 5:** Antimicrobial susceptibility testing of the MRSA isolate recovered from the initial blood culture. MIC values and susceptibility interpretations are reported as provided by the microbiology laboratory. MIC: minimum inhibitory concentration; MRSA: methicillin-resistant *Staphylococcus aureus*

Antimicrobial	MIC (µg/mL)	Interpretation
Amoxicillin/clavulanate	≤4/2	Resistant
Cefazolin	≤8	Resistant
Cefuroxime	≤8	Resistant
Clindamycin	≤0.5	Susceptible
Erythromycin	≤0.25	Susceptible
Oxacillin	>2	Resistant
Penicillin	2	Resistant
Tetracycline	≤4	Susceptible
Trimethoprim/sulfamethoxazole	≤0.5/9.5	Susceptible
Vancomycin	1	Susceptible

## Discussion

Pyomyositis is a bacterial infection of skeletal muscle characterized by suppurative inflammation and abscess formation [1,2]. Although historically associated with tropical regions, its incidence has increased in temperate climates, particularly among patients with diabetes mellitus, immunosuppression, chronic skin infections, chronic kidney disease, and intravenous drug use [3-5]. *Staphylococcus aureus*, including methicillin-resistant *S. aureus* (MRSA), remains the predominant causative organism [1,6]. Deep pelvic involvement is less commonly described and may present with nonspecific symptoms such as fever, bacteremia, hip pain, or difficulty ambulating [2-4,7-9]. In this patient, poorly controlled diabetes, peripheral neuropathy, and chronic diabetic foot ulcerations represented important predisposing factors.

MRI played an important role in defining the extent of the deep musculoskeletal infection. The Infectious Diseases Society of America recommends MRI as the preferred imaging modality for establishing the diagnosis of pyomyositis [1]. MRI findings may include muscle edema during early infection and rim-enhancing intramuscular collections with surrounding edema after abscess formation [10-12]. In this patient, MRI demonstrated intramuscular abscesses involving the obturator internus, gluteus minimus, and gluteus medius muscles, with extension through the greater sciatic notch and no evidence of pelvic osteomyelitis or septic arthritis. These findings provided more detailed characterization of the anatomic extent of infection than the initial CT examination.

Given the persistent MRSA bacteremia and progressive hip pain, alternative infectious sources and musculoskeletal diagnoses were considered. Infective endocarditis was excluded by negative transthoracic and transesophageal echocardiography. Pelvic MRI showed no evidence of septic arthritis or pelvic osteomyelitis, while lumbar MRI demonstrated degenerative changes without evidence of infection. Podiatric evaluation showed no clinical signs of acute foot infection, and foot radiographs showed no evidence of osteomyelitis. Although the MRSA-positive chronic foot ulcers remained a possible portal of entry, the multiple intramuscular pelvic abscesses identified on MRI supported pelvic pyomyositis as the principal deep infectious focus.

The obturator internus is an infrequently reported site of pyomyositis, with previous cases describing presentations associated with sciatica [7,9]. In this case, MRI demonstrated involvement of the obturator internus, gluteus minimus, and gluteus medius muscles, with extension through the greater sciatic notch and inflammatory changes along the expected course of the sciatic nerve. The greater sciatic foramen provides an anatomic communication between the pelvis and gluteal region and transmits the sciatic nerve and other neurovascular structures [13]. Previous reports have also described Gram-negative obturator muscle infection in a patient with diabetes and bilateral obturator pyomyositis [14,15]. The extent of inflammation along this region provides an anatomic correlate for the patient's radiating posterior leg pain.

The patient's chronic diabetic foot ulcers are an important consideration in interpreting the persistent MRSA bacteremia. MRSA was isolated from both foot wounds as well as serial blood cultures, while *Escherichia coli* was additionally isolated from the right foot ulcer. Although the precise route of dissemination cannot be established from the available clinical data, the chronic ulcerations provided a plausible source for MRSA bacteremia with subsequent hematogenous involvement of the deep pelvic musculature. This interpretation is particularly relevant given the patient's poorly controlled diabetes and peripheral neuropathy. Podiatric evaluation and debridement demonstrated no clinical signs of acute infection, and foot radiographs showed no findings suggestive of osteomyelitis; however, these findings do not exclude the chronic ulcers as a potential source of bacteremia.

Management was complicated by the location and distribution of the pelvic abscesses. Drainage of purulent material is recommended when feasible as part of the management of pyomyositis [1,3]. In this patient, general surgery and interventional radiology determined that the pelvic collections were not safely amenable to operative or percutaneous drainage. Therefore, management relied on prolonged targeted intravenous antimicrobial therapy and continued clinical and microbiologic monitoring.

Persistent MRSA bacteremia was treated initially with intravenous vancomycin, followed by daptomycin beginning on hospital day eight. Blood cultures remained positive through hospital day 10 and became negative on hospital day 11, three days after daptomycin initiation, with subsequent cultures remaining negative. The patient demonstrated clinical improvement and was discharged on hospital day 18 with daily outpatient intravenous daptomycin infusions to complete approximately seven weeks of antimicrobial therapy under Infectious Disease team supervision. The inability to drain the pelvic collections underscores the importance of prolonged antimicrobial therapy and close follow-up when definitive source control cannot be achieved.

This case demonstrates the clinical course of chronic diabetic foot ulcerations followed by persistent MRSA bacteremia and deep pelvic pyomyositis involving the obturator internus, gluteus minimus, and gluteus medius muscles with extension through the greater sciatic notch. In patients with persistent MRSA bacteremia and hip or radiating leg pain, evaluation for deep musculoskeletal infection should be considered, particularly when initial imaging does not fully characterize the extent of disease. MRI can provide detailed assessment of intramuscular abscesses and their extension into adjacent anatomic compartments.

## Conclusions

Deep pelvic pyomyositis should be considered in patients with persistent *Staphylococcus aureus* bacteremia, hip pain, and suspected deep musculoskeletal infection, particularly when earlier CT imaging is inconclusive. This case demonstrates *Staphylococcus aureus* infection involving the obturator internus, gluteus minimus, and gluteus medius muscles, with extension through the greater sciatic notch. MRI established the diagnosis and delineated the extent of infection.
